# Pneumonia treatment by photodynamic therapy with extracorporeal illumination ‐ an experimental model

**DOI:** 10.14814/phy2.13190

**Published:** 2017-03-14

**Authors:** Mariana C. Geralde, Ilaiáli S. Leite, Natalia M. Inada, Ana Carolina G. Salina, Alexandra I. Medeiros, Wolfgang M. Kuebler, Cristina Kurachi, Vanderlei S. Bagnato

**Affiliations:** ^1^ University of São PauloSão CarlosBrazil; ^2^ Federal University of São CarlosSão CarlosBrazil; ^3^ São Paulo State UniversityAraraquaraBrazil; ^4^ Keenan Research Centre of St. Michael's HospitalTorontoOntarioCanada

**Keywords:** Extracorporeal illumination, indocyanine green, photodynamic therapy, pneumonia, *Streptococcus pneumoniae*

## Abstract

Infectious pneumonia is a major cause of morbidity/mortality, mainly because of the increasing rate of microorganisms resistant to antibiotics. Photodynamic Therapy (PDT) is emerging as a promising approach, as effects are based on oxidative stress, preventing microorganism resistance. In two previous studies, the in vitro inactivation of *Streptococcus pneumoniae* using indocyanine green (ICG) and infrared light source was a success killing 5 log_10_ colony‐forming units (CFU/mL) with only 10 *μ*mol/L ICG. In this work, a proof‐of‐principle protocol was designed to treat lung infections by PDT using extracorporeal illumination with a 780 nm laser device and also ICG as photosensitizer. Hairless mice were infected with *S. pneumoniae* and PDT was performed two days after infection. For control groups, CFU recovery ranged between 10^3^–10^4^/mouse. For PDT group, however, no bacteria were recovered in 80% of the animals. Based on this result, animal survival was evaluated separately over 50 days. No deaths occurred in PDT group, whereas 60% of the control group died. Our results indicate that extracorporeal PDT has the potential for pneumonia treatment, and pulmonary decontamination with PDT may be used as a single therapy or as an antibiotics adjuvant.

## Introduction

Nosocomial infections are usually caused by resistant pathogens and occur in hospitals worldwide, with high index of morbidity, mortality, and hospital costs (Sopena et al. 2014). Microorganisms can infect the lower respiratory tract, which is considered largely sterile in healthy individuals, by four different mechanisms (Nair and Niederman 2013): (1) aspiration of secretions containing pathogens from oropharynx, gastric cavity, or nasal cavities; (2) spread of bacteria from a nearby area, such as pleura; (3) aspiration through respiratory therapy devices and inhalation of contaminated aerosols; (4) hematogeneous translocation into lungs from systemic infection sites. In hospitals, intubation and mechanical ventilation are commonly followed by infection (“ventilator‐associated pneumonia”), which is considered the main complication for ventilated patients.

In developing countries, the main etiological agents causing pneumonia are bacteria, mostly *Streptococcus pneumoniae* (30–50%). The second most common agent is *Haemophilus influenzae* type b (Hib), followed by *Staphylococcus aureus* and *Klebsiella pneumoniae*(Singh and Aneja 2011).

Community‐acquired pneumonia (CAP) is the major cause of children illnesses – 20% of child deaths worldwide (Berezin et al. 2012). In Brazil, during 2007, pneumonia was the principal non‐obstetric cause of hospitalization, accounting for close to one million cases (Corrêa et al. 2009). According to the World Health Organization (WHO), pneumonia incidence is around 450 million cases every year, with approximately 4 million deaths per year. It is responsible for the deaths of about 1 million children under five years‐old yearly (WHO, 2015). In the USA, the estimated annual number of CAP episodes in adults is approximately 5.2 million, with the highest incidence among those aged 65 years and older (Jiang et al. 2013).

These numbers describe a present situation that is prone to get worse rather than better because of the aging profile of the population in Western countries, and to the development of single‐ and multi‐drug resistance in infectious pathogens. As a result, there is a need for alternative or adjunct treatments to reduce morbidity and mortality rates.

Photodynamic therapy (PDT) consists in the use of a photosensitizer (PS) which, following activation by light at a PS‐specific wavelength, will react with molecules in its microenvironment. The resulting reaction can occur by transfer of hydrogen or electrons, leading to the formation of free radicals, reactive oxygen species (ROS) (type‐I reaction) or by energy transfer to molecular oxygen (type‐II reaction), producing mainly singlet oxygen. Both reactions can kill microorganism almost immediately by oxidative damage (Mantareva et al. 2011; Rozenbaum et al. 2015).

PDT of microorganisms depends on a higher concentration of PS at the target cells, for example, bacteria, compared with the surrounding host tissues. This is essential to promote a relatively selective toxic effect on the microorganisms when compared with the host parenchyma (Maisch 2007).

Notably, PDT is already used clinically as treatment for several different infections disease, such as skin ulcers, oral candidiasis, periodontal diseases, acne vulgaris and many others (Christodoulides et al. 2008; Scwingel et al. 2012; Lei et al. 2015; Moftah et al. 2016; Yang et al. 2016).

Indocyanine green (ICG) is a tricarbocyanine, an organic dye that has been used clinically for diagnostic purposes since 1956 in several applications (Fickweiler et al. 1997; Steinbrink et al. 2006; Yaseen et al. 2009), approved by the USA Food and Drug Administration in 1959. Is amphiphilic and soluble in inorganic solvents such as Dimethylsulfoxide (DMSO) and methanol, as well as in aqueous media, including phosphate‐buffered saline (PBS) and cell culture medium. The absorption peak of ICG is at 805 nm (near‐infrared) where penetration into biological tissues is higher because of low absorption by hemoglobin, melanin, and water, which makes ICG an ideal photosensitizer for deep‐tissue PDT (Urbanska et al. 2002; Crescenzi et al. 2004) applications, which require light penetration through the parenchyma. In two previous studies of our group, we have successfully demonstrated the efficacy of PDT with ICG killing *S. pneumoniae* in the same experimental condition where alveolar macrophage viability was preserved (Leite et al. 2014).

Other authors is indicating the use of ICG for treatment of several cancer types, acne vulgaris, in occlusion of choroidal neovascularization, and in eradicating microorganisms present in wounds and burns (Bäumler et al. 1999; Costa et al. 2001; Tuchin et al. 2003; Omar et al. 2008; Lim and Oh 2011; Montazerabadi et al. 2012).

Based on the dire need for novel therapies for pneumonia, and the promising results obtained with PDT in other infectious diseases, we hypothesized that PDT could be an alternative or adjunct therapy for pneumonia treatment. To this end, we evaluated the efficacy of PDT with extracorporeal illumination using an infrared light source and ICG as PS for the treatment of pneumonia in an experimental mouse model.

## Material and Methods

### Chemicals and reagents

Xylazine and Ketamine were purchased from Sespo‐Ceva Santé Animale (Paulínia, São Paulo, Brazil). Indocyanine green – ICG was obtained from Ophthalmos, São Paulo – Brazil. Dimethylsulfoxide (DMSO), ethanol, sodium chloride, potassium chloride, dibasic sodium phosphate, monobasic potassium phosphate were obtained from Sigma‐Aldrich (St. Louis, Missouri).

### Animal model

Animal experiments were approved by the Animal Ethics Committee of School of Pharmaceutical Science, UNESP ‐ São Paulo State University (number 46/2013, approved on August 15th, 2013). Eight‐week‐old female SKH‐1 hairless mice (*n* = 28) were used. For induction of lung infection, animals were anesthetized by intraperitoneal administration of Xylazine (0.1 mg/kg) and Ketamine (100 mg/kg) and then, the total amount of 10^8^ *Streptococcus pneumoniae* (ATCC 49619) bacteria (kindly provided by Dr. Medeiros from Immunology Laboratory, São Paulo State University) were suspended in phosphate‐buffered saline (PBS) and instilled into the nostrils of the animals using a micropipette. The total volume of the instilled inoculum was 30 *μ*L per animal (15 *μ*L per nostril).

Animals were randomly divided into 4 experimental groups: control (no treatment), light‐only (extracorporeal illumination), photosensitizer‐only (ICG) group, and full photodynamic therapy (PDT) with ICG and extracorporeal illumination.

### Treatments

Before any treatment protocol, animals were anesthetized again using the same protocol as the one described for the infection. For the PDT group, 1 mg of ICG was dissolved in 100 *μ*L of sterile DMSO and diluted with 900 *μ*L of PBS immediately before instillation into the nostril of the animal. ICG was used at a concentration of 100 *μ*mol/L and 15 *μ*L were instilled in each nostril.

**Figure 1:**
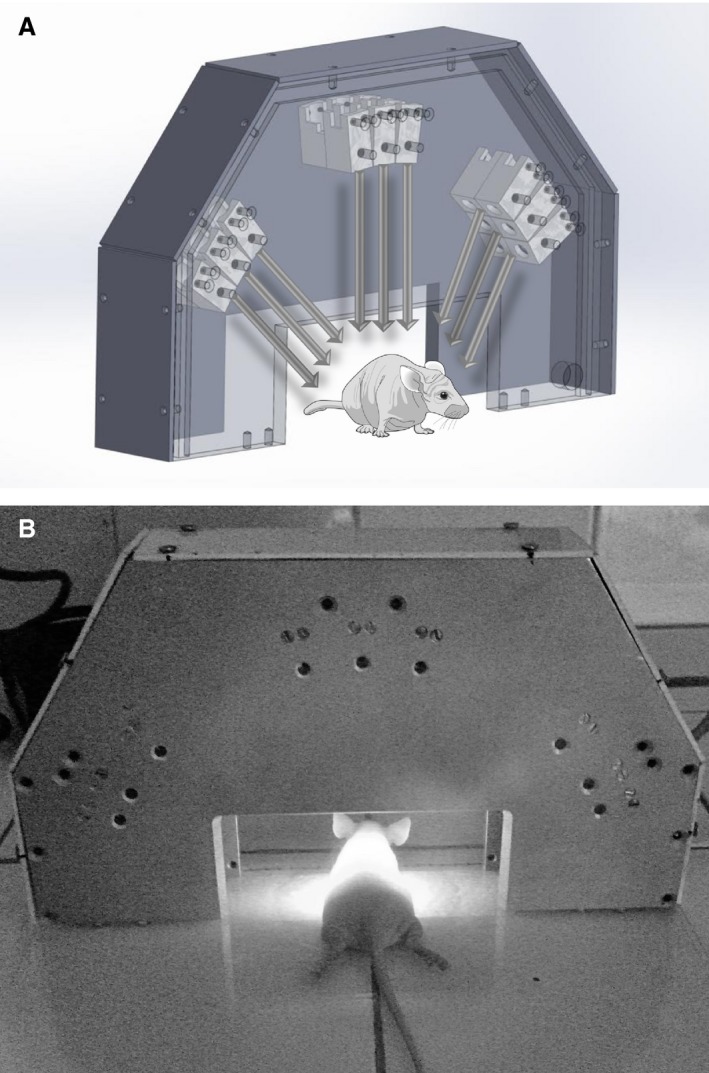
(A) Custom‐made illumination device contending 18 laser diodes generating illumination of the animal's thorax at 780 nm. The arrows illustrate the laser beam propagation. (B) Animal positioning during the irradiation procedure.

Three minutes after the instillation, the animal was positioned inside a custom‐made laser device containing 18 laser diodes emitting monochromatic light at 780 nm. For illumination, an irradiance of 60 mW/cm^2^ and a total dose of 120 J/cm^2^ was delivered onto the animal dorsum. The custom‐built device for this experiment allows the animal placement in the prone position during irradiation as shown in Figure 1.

For the light‐only group, the same procedures as in the PDT group were performed, yet without administration of ICG. For the PS only group, animals were instilled with ICG, but not exposed to subsequent illumination.

### Colony‐forming‐units recovery

Recovery of colony‐forming units (CFU) was performed to evaluate the effectiveness of PDT in mouse lungs with or without PDT treatment (*n* = 4–5 each group). Seven days after PDT treatment or respective controls, animals were euthanized and their lungs were removed and homogenized with 1 mL of sterile PBS, then ten‐fold serial dilutions in sterile PBS of each sample were prepared and spread on blood‐agar plates. CFU were counted after 24 h of incubation at 37°C and 5% CO_2_.

### Survival rate

For survival experiments, hairless female mice (*n* = 10 in total) were infected with *S. pneumoniae* as described previously, and 2 days after infection animals were subjected to either PDT or no treatment. Animals were monitored for 50 days.

### Statistical analysis

The result of any triple group was expressed as mean ± standard error of measurement. Student's *t*‐tests were performed to evaluate whether two triple groups were significantly different from each other. Analyses were carried out using the Graphpad Prism software. Statistical significance was defined at *P* < 0.05 level (95% confidence level).

## Results

**Figure 2:**
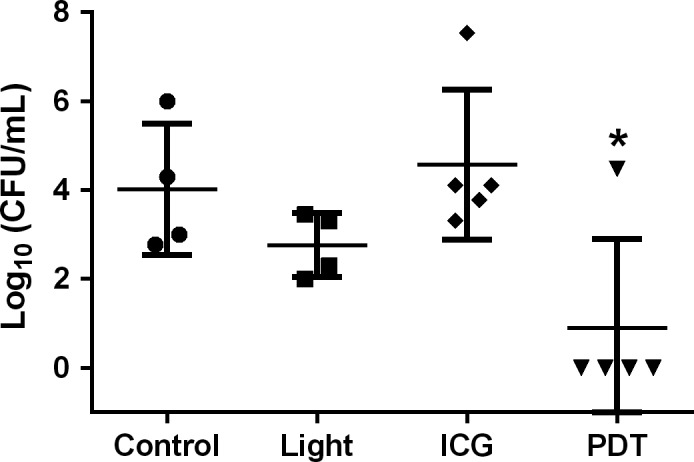
Scatter plot and mean ± SD of CFU recovery for mice infected with 10^8^ *S. pneumoniae* cells and treated with only PBS instillation (Control), PBS instillation followed by 120 J/cm^2^ of light dose (Light), only 100 *μ*mol/L of indocyanine green instillation (ICG), or a combination of 100 *μ*mol/L of indocyanine green instillation and 120 J/cm^2^ of light dose (PDT). * *P *˂ 0.05 versus Control, test Mann‐Whitney, *n* = 4–5 each. CFU, colony‐forming units; ICG, indocyanine green; PBS, phosphate‐buffered saline.

For control (no treatment), light‐only and ICG‐only groups, CFU recovery ranged on average between 10^3^ and 10^4^. For the PDT group, however, no bacteria were recovered in 80% of the animals. Hence, PDT reduced the recovery of CFU by approximately three orders of magnitude (Fig. 2), thus providing proof‐of‐principle for the validity and efficiency of the proposed concept of bacterial decontamination by PDT in pneumonia.

Notably, a small decrease in CFU was also observed in infected mice treated with light alone as compared to untreated controls, which however did not reach the level of significance. This effect may potentially be attributable to light effects which have been shown to stimulate the immune system (Møller et al. 2005; Aranow 2011).

**Figure 3:**
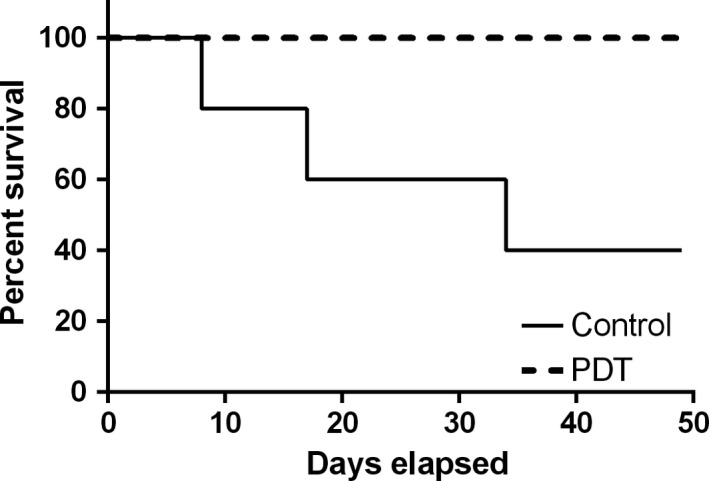
Kaplan‐Meier curve shows the survival of untreated control mice and mice treated with PDT (120 J/cm^2^ of light + 100 *μ*mol/L of ICG) over 50 days post‐infection with 10^8^ *S. pneumoniae* cells. Treatment was performed 2 days after the bacterial inoculation. In the control group, 60% of animals (3 out of 5) died within 32 days following infection. However, no animals died in the Photodynamic Therapy group over the 50 days following infection.

Based on the results of the colony‐forming units recovery, we next performed experiments to assess the effects of PDT on survival in the murine pneumonia model. Over the period of 50 days post‐infection, 60% of the control group (infected animals without treatment) died. In contrast, no deaths were observed over the same period in the PDT group, as shown in the Kaplan‐Meier curve in Figure 3. Only the PDT and the Control group were evaluated because no difference between the ICG, Control, and Light groups were observed in the first experiment (Colony‐forming‐units recovery after 7 days).

These data are the first to demonstrate a survival benefit of PDT in experimental pneumonia, and provide thus important proof‐of‐principle for the therapeutic potential of PDT for the treatment of lung infections including pneumonia.

## Discussion

Our findings are in line with a series of previously published data by our group on the effectiveness of PDT in bacterial decontamination, and extend its use to PDT by extracorporeal illumination in cases of pneumonia. In 2014, we reported an in vitro inactivation of *S. pneumoniae* by PDT, with a combination of light at 850 nm and 10 *μ*mol/L ICG (Leite et al. 2014). In this study, was evaluated the effect of PDT on alveolar macrophages, which are key phagocytes in the innate immune defense of the lung. Importantly, alveolar macrophage viability was above 90% following PDT with ICG and light at 850 nm suggesting that PDT treatment is safe and does not harm the host immune system. These results also pointed toward the great potential of ICG as a photosensitizer. A different wavelength (780 nm laser device) was tested to compare the ICG efficiency to be photoactivated for two different light sources and wavelengths. And in conclusion, it is possible to optimize the ICG concentration, incubation time, and light dose in a way to obtain bacterial death in the absence of toxic effects on the co‐incubated macrophages (Leite et al. 2014). While these in vitro results point out to a potential therapeutic usefulness of PDT for bacterial decontamination, the present in vivo results now provide for the first time evidence for the feasibility and effectiveness of this approach in a live animal model.

An important review was published discussing if the ICG is a photosensitizer or a chromophore. Independently of the light source used, is reported that ^1^O_2_ (singlet oxygen) but is not detectable (Giraudeau et al. 2013). It is also known that the positive characteristic of ICG of lower systemic toxicity occurs because of the rapid elimination by the hepatobiliary system (Giraudeau et al. 2013). The cytotoxicity has a median lethal dose with a LD_50_ of 50–80 mg/kg (Taichman et al. 1987).

A critical general issue in the therapeutic use of PDT is the effective delivery of light. While PDT has been highly successful for the treatment of cancers and infectious and inflammatory diseases on the body′s skin or mucosa, which are easily accessible for extracorporeal illumination, the situation is more complex for internal organs such as the lung because of the limited capacity of tissue penetration by light. While extracorporeal illumination with near‐infrared and infrared light remains a challenge, it bears a huge potential to increase the use of PDT, specifically for infectious diseases of deeper tissues and organs. In 2014, we were able to report important advances for the use of extracorporeal illumination. In a liquid phantom and by measuring light transmittance from an 810 nm laser in a *post‐mortem* model we could show that the light can pass through the mouse chest albeit with a reduced intensity (Geralde et al. 2014). These results led us to propose that infrared light may be used effectively for extracorporeal illumination in PDT. Based on this concept, we performed a series of light penetration experiments in *post‐mortem* mice with 780 nm lasers, which indicated that approximately 50% of the light is absorbed and/or scattered in tissue (data not shown). From these experiments, we determined the extracorporeal applied light dose of 120 J/cm^2^ in order to achieve a dose of 60 J/cm^2^ in the lung.

The use of PDT with ICG and infrared light for the treatment of infections is further validated by a recent case report. In this report, the authors treated a verrucous epidermal nevus (VEN) with PDT using ICG as a photosensitizer and intense pulsed light (IPL) at a wavelength of 500–800 nm. After 6 sessions the reported cosmetic and the clinical responses were considered excellent, and no recurrence was observed over a follow‐up of 2 years (Kim et al. 2015). Similarly, Genina et al. (2004) used ICG as a photosensitizer and infrared light (830 nm) for the treatment of acne. The investigators performed two different treatment regimes, one with a single PDT session and the other with eight sequential treatments. After one month, they observed that only the multiple treatments reduced inflammation and improved the state of the skin (Genina et al. 2004), whereas the single treatment had no effect. Hence, repetitive treatment may analogously provide additional benefits in experimental and clinical pneumonia, a notion that we hope to test in future experiments.

Topaloglu et al. (2013) concluded that the output power up to 1 W of a diode laser emitting at 809 nm did not cause any photothermal effect during PDT applications in vitro using a Gram negative bacteria *Pseudomonas aeruginosa* (ATCC 27853). The mechanisms of how ICG kills bacteria are not clear but is possible that has a combination of several effects such as via reactive oxygen species production plus thermal effects (Giraudeau et al. 2013).

Taken together, these findings confirm the potential of PDT to eliminate bacteria and treat infectious diseases by extracorporeal illumination with infrared light following the administration of the PS ICG. Our present results demonstrate the first evidence for the feasibility and effectiveness of this technique for the treatment of lung infections, a concept that we aim to further develop by rigorous testing of different photosensitizer incubation times and doses of delivered light in an attempt to optimize parameters for safe in vivo application.

As pneumonia and other infections remain and because of the development of bacterial resistances increase to present a life‐threatening problem in hospitalized patients, there is a rapidly growing need for novel techniques for bacterial decontamination. PDT is a well‐established technique for the elimination of microorganisms by direct illumination. Here, we demonstrate the applicability of this principle also in cases when illumination has to overcome barriers composed of healthy tissue such as for the treatment of lung infections.

The reported reduction in CFU by several orders of magnitude through the use of PDT demonstrates the feasibility of extracorporeal illumination PDT and opens up a new range of opportunities. Furthermore, we provide evidence that extracorporeal PDT can indeed generate a functional survival benefit in experimental pneumonia. Based on the present proof‐of‐principle, we can now take the next steps to assure secure application and optimize treatment parameters for extracorporeal PDT before the start of first clinical applications.

## Conflict of Interest

None declared.
